# microRNAs: important regulators of stem cells

**DOI:** 10.1186/s13287-017-0551-0

**Published:** 2017-05-11

**Authors:** Na Li, Bo Long, Wei Han, Shumin Yuan, Kun Wang

**Affiliations:** 1 State Key Laboratory of Biomembrane and Membrane Biotechnology, Institute of Zoology, Chinese Academy of Sciences, Beijing, 100101 China; 2 Beijing Anzhen Hospital, Capital Medical University, 2 Anzhen Road, Chaoyang District, Beijing, 100029 China; 3 Guilin Medical University, Guilin, Guangxi 541004 China

**Keywords:** Stem cell, miRNAs, Reprogramming, Pluripotency, Differentiation

## Abstract

Stem cells are undifferentiated cells and have multi-lineage differentiation potential. Generally, stem cells are classified into adult stem cells, embryonic stem cells (ESCs) and induced pluripotent stem cells (iPSCs). Stem cells have great potential in clinical therapy due to their pluripotency and self-renewal ability. microRNAs (miRNAs) are small non-coding RNAs which are evolutionarily conserved and participate in the pathogenesis of many diseases, cell cycle regulation, apoptosis, aging, cell fate decisions, and different signaling pathways. Different kinds of stem cells possess distinct miRNA expression profiles. Our review summarizes the critical roles of miRNAs in stem cell reprogramming, pluripotency maintenance, and differentiation. In the future, miRNAs may greatly contribute to stem cell clinical therapy and have potential applications in regenerative medicine.

**Electronic supplementary material:**

The online version of this article (doi:10.1186/s13287-017-0551-0) contains supplementary material, which is available to authorized users.

## Background

Stem cells are undifferentiated cells and have multi-lineage differentiation potential. Generally, stem cells are classified into adult stem cells, embryonic stem cells (ESCs) and induced pluripotent stem cells (iPSCs). Adult stem cells are named according to the tissue in which they originated, such as mesenchymal stem cells (MSCs), hematopoietic stem cells (HSCs), cardiac stem cells, neural stem cells (NSCs), endothelial stem cells, etc. ESCs are pluripotent stem cells derived from the inner cell mass of a blastocyst or earlier morula stage embryos in epiblast tissue [1], whereas iPSCs are directly generated through somatic cell reprogramming [2]. Stem cells have great potential in clinical therapy due to their pluripotency and self-renewal ability. In preclinical animal experiments, cardiovascular cells [3], neural cells [4], and osteoblasts [5] differentiated from stem cells have been transplanted to repair damaged organs.

microRNAs (miRNAs) are small non-coding single-stranded RNAs with a length of about 21–22 nucleotides that repress gene expression through translational inhibition or by promoting the degradation of mRNAs. In the nucleus, the transcribed primary miRNAs (pri-miRNAs) are cleaved to form precursor miRNAs (pre-miRNAs) containing short hairpins by the microprocessor, which is composed of RNA-binding protein DGCR8 and RNase III enzyme Drosha. Pre-miRNAs are then exported into the cytoplasm by exportin-5 and finally processed by Dicer to form mature miRNAs [6]. Mature miRNAs are incorporated into the RNA-induced silencing complex (RISC) to regulate gene expression post-transcriptionally by base pairing with the 3′ untranslated region (3′ UTR) of their targets [7]. However, a few miRNAs have been demonstrated to repress gene expression by binding the open reading frame region of the target [8]. Previous studies have demonstrated that miRNAs play critical roles in physiological processes and the pathogenesis of many diseases. In general, miRNAs have great potential for treating various diseases, such as myocardial injury and neurodegenerative, blood, and muscle diseases [9–12]. Recently, miRNAs are discovered to be important regulators of stem cells. Genetically ablated *Dicer* ^−/−^ or *Dgcr8* ^−/−^ ESCs show abnormal differentiation [13, 14]. Studies show that miRNAs regulate the state of stem cells by directly targeting the 3′ UTR of pluripotency factors. For instance, miR-145 represses the pluripotency of human ESCs through targeting octamer-binding transcription factor 4 (*Oct4*; also known as Pou5f1, POU domain, class 5, transcription factor 1), sex determining region Y-box 2 (*Sox2*), and kruppel-like factor 4 (*Klf4*) [15]. In addition, miRNAs target the coding regions of transcription factors to modulate stem cell differentiation. miR-296, miR-470, and miR-134 regulate mouse ESC differentiation by targeting the coding regions of *Nanog*, *Oct4*, and *Sox2* [16]. Other classified miRNAs also regulate the fate of stem cells. Embryonic stem cell-specific (ESCC) miRNAs (also called ESC-specific cell cycle-regulating miRNAs), c-Myc-induced miRNAs, p53-targeting miRNAs, and early embryonic miRNA cluster (EEmiRC) also regulate the self-renewal, reprogramming and differentiation of stem cells [17–20].

## miRNAs and stem cell reprogramming

### Cell reprogramming

Cell reprogramming refers to the process of a differentiated somatic cell being reprogrammed into a pluripotent state or even forming a new individual under particular conditions. Cell reprogramming involves nuclear transplantation and iPSC reprogramming technologies. Nuclear transplantation forms a new individual through transferring a donor somatic nucleus into an enucleated oocyte. The iPSC technologies are used to reprogram somatic cells into pluripotent states through enhanced expression of pluripotency-related genes or proteins [2, 21]. Our review focuses on somatic cell reprogramming.

Somatic cell reprogramming was discovered in 2006. iPSCs are successfully generated from mouse fibroblasts through virus-mediated transfection of *Oct3/4*, *Sox2*, *Klf4*, and *c-Myc* [2]. Human iPSCs are generated by transduction of the alternative combinations of *Oct3/4*, *Sox2*, *Nanog*, and *Lin28* [22]. However, the reprogramming efficiency is about 0.02–0.08% for virus-mediated transduction of pluripotent genes. Since the virus can integrate into the genome randomly, this method carries a high risk of tumorigenicity. Recently, lower tumorigenic iPSCs have been generated. For example, mouse iPSCs were generated through transfection of two plasmids, one containing the complementary DNAs (cDNAs﻿﻿)﻿ of *Oct3/4*, *Sox2*, and *Klf4*, and the other containing the *c-Myc* cDNA. However, the reprogramming efficiency was substantially lower than with the virus-free method [23]. Furthermore, synthetically modified mRNA has been used to generate human iPSCs more efficiently. The reprogramming efficiency is about 1.4% with lower tumorigenicity potential [24].

### miRNAs participate in the regulation of stem cell reprogramming

miRNAs regulate the reprogramming efficiency of iPSCs. The ESCC miRNAs enhance reprogramming efficiency. For instance, over-expression of the miR-290 family or miR-302 family enhances reprogramming efficiency [25]. Human miR-372 (an ortholog of the mouse miR-290 cluster and miR-302 cluster), the miR-17-92 cluster, the miR-106b-25 cluster, and the miR-106a-363 cluster (sharing very similar seed sequence with the miR-302 cluster) have been proven to increase reprogramming efficiency [26, 27]. Strikingly, miRNAs can reprogram somatic cells into iPSCs directly. For example, the miR-302 cluster can reprogram human skin cancer cells into a pluripotent state [28]. Also, direct transfection of the mature double-stranded miR-200c, miR-302, and miR-369 family can reprogram mouse and human somatic cells into pluripotent states [29]. miRNA-induced iPSCs have a reprogramming efficiency above 10% and also the lowest tumorigenicity.

Autologous iPSCs can be directly obtained through somatic cell reprogramming and have potential applications in regenerative medicine. Furthermore, use of autologous iPSCs can avoid ethical issues and immunological rejection. However, autologous iPSCs are still assocaited with lower reprogramming efficiency and tumorigenicity. In the future, autologous iPSCs may have great value in regenerative medicine.

### Mechanism of miRNA-mediated stem cell reprogramming

miRNAs regulate stem cell reprogramming by regulating the reprogramming process. This process has been divided into three phases: initiation, maturation, and stabilization [30]. The initiation phase exhibits a mesenchymal-to-epithlial transition (MET) character. The miR-200 family (miR-200b and miR-200c), the miR-106a-363 and miR-302-367 cluster, and miR-93/106b, which are activated by OSK (Oct4, Sox2, Klf4) or OSKM (Oct4, Sox2, Klf4, c-myc), can promote the MET process during iPSC initiation [27, 30, 31]. Also, down-regulated miR-30/let-7 family and up-regulated miR-17, miR-19, miR-290, and miR-8 family miRNAs play important roles in the activation and maintenance of pluripotency [30].

Furthermore, miRNAs participate in the reprogramming process by regulating cell cycle factors. For instance, the miR-25 family and miR-130/301/721 family target p21, a cell cycle inhibitor, to promote reprogramming efficiency [27, 32]. Depletion of miR-34a significantly promotes the somatic cell reprogramming process. Studies have found that miR-34a and p21 together regulate reprogramming efficiency by targeting p53 [33]. The regulatory function of miRNAs on stem cell reprogramming is depicted in Additional file 1.

### miRNAs and stem cell pluripotency

miRNAs modulate the pluripotency of stem cells, generally regulating the pluripotency factors by directly targeting their 3′ UTRs. miR-145 represses the pluripotency of human ESCs by repressing the expression of *Oct4*, *Sox2*, and *Klf4* [15]. However, miR-134, miR-296, and miR-470 target the coding sequence of *Sox2*, *Nanog*, and *Oct4* to regulate the pluripotency of ESCs [16]. A recent study has shown that the miR-290 family affects the pluripotency and differentiation of ESCs through epigenetic regulation of de novo DNA methylation [34]. Another study has shown that *Oct4*, *Sox2*, *Nanog*, and Transcription factor 3 (*Tcf3*) have binding sites in the promoter region of most miRNAs that are preferentially or exclusively expressed in ESCs. These transcription factors also regulate the expression of miRNAs [35].

### miRNAs and stem cell differentiation

Stem cells possess specific miRNA expression profiles which modulate stem cell fate [35]. This mechanism could be used to terminally differentiate cells from stem cells in order to treat various diseases, including myocardial infarction, neurodegenerative diseases, blood diseases, and muscle diseases.

### miRNAs and cardiovascular differentiation

miRNAs modulate cardiovascular differentiation of cardiomyocyte progenitor cells and stem cells, including the differentiation of cardiomyocytes, vascular smooth muscle cells (SMCs), and endothelial cells (ECs). On one hand, miR-499 enhances cardiovascular differentiation of human-derived cardiomyocyte progenitor cells by targeting Sox6 [36]; on the other hand, miRNAs regulate the cardiovascular differentiation of ESCs and iPSCs. In the infarcted heart, miR-1 promotes cardiac differentiation of ESCs and inhibits cardiomyocyte apoptosis through the phosphatase and tensin homology deleted on chromosome ten (PTEN)/Akt pathway [37]. miR-1 also promotes SMC differentiation of retinoid acid-induced ESCs by targeting Klf4 [38]. It has been demonstrated that retinoic acid promotes nuclear translocation of NF-κB, which activates the miR-10a expression; miR-10a promotes SMC differentiation from mESCs by targeting histone deacetylase 4 (HDAC4) [39]. Kim and colleagues have shown that miR-6086 and miR-6087 block EC differentiation of human ESCs by inhibiting the expression of CDH5 and endoglin, respectively [40]. In mouse iPSCs, miR-199b promotes EC differentiation by modulating signal transducer and activator of transcription 3 (STAT3)/vascular endothelial growth factor (VEGF) signaling [41]. Depletion of miR-495 promotes EC differentiation and angiogenesis through targeting vascular endothelial zinc finger 1 (VEZF1) in human iPSCs [42]. miRNA-mediated cardiovascular differentiation of stem cells has great therapeutic value in regenerative medicine. The specific function of miRNAs in cardiovascular differentiation is summarized in Additional file 2.

### miRNAs and neural differentiation

Previous studies have shown that miRNAs also play critical roles in neurogenesis. Doetsch and colleagues reported that miR-124 promotes the neural differentiation of the subventricular zone, which is the largest neurogenic niche in adult mammalian brain [43]. Forced expression of miR-34a results in reduction of dendritic length, neuron branch numbers, and the numbers of functional synapses and disruption of inhibitory inputs [44]. miRNAs also regulate NSC differentiation through regulatory loops. miR-9 promotes NSC differentiation by targeting the nuclear receptor TLX, which inhibits the expression of pri-miR-9. TLX and miR-9 form a negative regulatory loop balancing the proliferation and differentiation of NSCs [45]. Furthermore, acute deficiency of Methy-CpG binding protein1 (MBD1) results in increased miR-184, which directly targets the brain development regulator Numblike (Numbl). MBD1-miR-184-Numbl forms a loop to regulate adult NSC differentiation [46].

More importantly, miRNAs regulate the neurogenesis of ESCs and iPSCs through targeting relative neural differention genes. miR-371-3 is highly expressed in human iPSCs and ESCs. Suppression of miR-371-3 promotes neural differentiation [47]. Down-regulation of miR-132 promotes the differentiation of tyrosine hydroxylase-positive neurons by suppressing Nurr1 [48]. Knockdown of Smad4 in human ESCs increases the percentage of the neural lineage commitment. miR-125, which is activated by inhibition of both the activin and BMP-dependent pathways, can promote human ESC differentiation towards the neural lineage through suppression of Smad4 [49]. NR2F2 (nuclear receptor subfamily 2, group F, member 2) is necessary for neural differentiation in human ESCs. *Oct4* and miR-302 can form a regulation loop during neural differentiation by interacting with NR2F2 [50]. The function of miRNAs in neural differentiation is illustrated in Additional file 3.

### miRNAs modulate osteogenic and chondrogenic differentiation of stem cells

Regeneration of osteogenic and chondrogenic cells shows great medical research value. Substantial progress has been made in generating osteogenic and chondrogenic cells from adult stem cells. miRNAs regulate the osteogenic and chondrogenic differentiation through targeting significant transcriptional factors and relative pathways during skeletal development. The ERK-dependent pathway plays a critical role in osteoblast differentiation. It can activate the phosphorylation of runt related transcription factor 2 (RUNX2), promote *Osterix* expression, and improve the activity of alkaline phosphatase. The focal adhesion kinase (FAK) is linked with the activation of ERK1/2 through extracellular matrix proteins. miR-138 suppresses differentiation of human MSCs into osteoblasts by directly targeting FAK and downstream signaling [51]. miR-23b induces chondrogenic differentiation of human MSCs by suppressing protein kinase A (PKA) signaling [52]. Over-expression of miR-335-5p significantly promotes the chondrogenic differentiation of mouse MSCs by targeting Dishevelled-associated activator of morphogenesis 1(Daam1) and rho-associated coiled-coil containing protein kinase 1 (ROCK1) [53]. Mechanisms by which miRNAs regulate the osteogenic and chondrogenic differentiation of stem cells is summarized in Additional file 4.

### miRNAs regulate hematopoietic differentiation of stem cells

miRNAs are key regulators of hematopoiesis in mammalians. Erkeland and colleagues discovered that ectopic expression of AAAGUGC seed-containing miRNAs enhance the primary hematopoietic progenitors [54]. Bartel and colleagues were the first to show that miR-181, miR-223, and miR-142 cloned from mouse bone marrow are preferentially expressed in hematopoietic tissues. miR-181 significantly promotes B-lymphocyte differentiation [55]. miRNAs also play a significant role during the proliferation and differentiation of HSCs. miR-125a is conservatively expressed in long-term HSCs and can increase the number of HSCs by targeting the apoptosis factor Bax1 [56]. Furthermore, overexpression of miR-125b leads to lethal myeloid leukemia in mice [57].

## Conclusions

Stem cells have great clinical value. Adult stem cells are currently used in clinical tissue and organ repair, which having the advantages of broad sources, no transplant rejection and lower tumorigenicity. ESCs have the ability to differentiate into various types of cells but applications are limited because of higher tumorigenicity and ethical restrictions. iPSCs can provide autologous somatic cells for transplantation that avoid the problems of ethical issues and transplant rejection. Recently, miRNA-mediated reprogramming technology has been discovered. miRNA-induced iPSCs have advantages of higher reprogramming efficiency and lower tumorigenicity. Although no clinical therapy using them has been performed so far, they may greatly aid the development of regenerative medicine in the future. We believe that stem cells will be highly beneficial for regenerative medicine.

miRNAs play critical roles in reprogramming process, pluripotency maintenance, and differentiation of stem cells. Our work focuses on the regulatory function of miRNAs in adult stem cells, ESCs, and iPSCs (Table 1). miRNAs regulate stem cell fate through targeting specific pluripotency factors or differentiation pathways. However, the exact mechanisms of the regulatory functions of miRNAs need to be elucidated and whether other molecules or mechanisms are involved remains to be explored. Moreover, the significant function of miRNAs in the determination of stem cell fate indicates the way miRNAs regulate mammalian development in vivo [58]. miRNAs may be developed as therapeutic targets. Whether mimics or antagomirs of specific miRNAs can be developed as treatments still needs further studies. For example, suppression of miR-371-3 could be used to promote neural differentiation. However, whether the miR-371-3 antagomir could cure neurodegenerative diseases in vivo needs to be elucidated. Research on the regulatory functions of miRNAs will contribute to stem cell-based clinical therapy and also to potential applications in regenerative medicine.

**Table 1:** miRNAs involved in the regulation of stem cell fate

mRNA	Function	Target/pathway	Reference
*miRNA-29a*	Regulates chondrogenic differentiation and cartilage formation	FOXO3A	[8]
*microRNA-145*	Represses pluripotency in human ESCs	*Oct4*, *Sox2, Klf4*	[15]
miR-296, mir-470 and miR-134	Regulate mouse ESC differentiation	*Nanog*, *Oct4*, *Sox2*	[16]
miR-290 family or miR-302 family	Enhances reprogramming efficiency	*Oct4*, *Sox2*, *Klf4*	[25]
Human miR-372	Improves reprogramming efficiency		[26]
miR-17-92 cluster, miR-106b-25 cluster, miR-106a-363 cluster	Improve reprogramming efficiency	TGF-βR2, p21	[27]
miR-302 cluster	Induces the pluripotent state in human skin cancer cells		[28]
Mature double-stranded miR-200c plus miR-302 and miR-369 family	Reprogram mouse and human somatic cells into pluripotent states		[29]
miR-200 family, miR-106a-363 and miR-302-367 cluster, miR-93/106b	Promote the MET process of the iPSC initiation phase		[27, 30, 31]
Down-regulated miR-30/let-7 family	Activate the pluripotent state		[30]
Up-regulated miR-17, miR-19, miR-290 and miR-8 family	Maintain the pluripotent state		[30]
miR-25 family and the miR-130/301/721 family	Promote reprogramming efficiency	p21	[27, 32]
miR-34a	Regulates reprogramming efficiency with p21	p53	[33]
miR-290 family	Regulates pluripotency and differentiation	Epigenetic regulation of de novo DNA methylation	[34]
miR-1	Promotes the cardiovascular differentiation of ESCs and inhibits cardiomyocyte apoptosis	PTEN/Akt pathway	[37]
miR-1	Promotes SMCs differentiation of retinoid acid-induced ESCs	Klf4	[38]
miR-10a	Promotes differentiation of mouse ESCs into SMCs	HDAC4	[39]
miR-6086 and miR-6087	Block differentiation of human ESCs into ECs	CDH5, endoglin	[40]
miR-199b	Promotes EC differentiation		[41]
Depletion of miR-495	Promotes EC differentiation and angiogenesis of human iPSCs	VEZF1	[42]
miR-9	Promotes NSC differentiation	Balancing the proliferation and differentiation of NSCs with TLX	[45]
miR-184	MBD1-miR-184-Numbl form a loop to regulate adult NSC differentiation	Numbl	[46]
Suppression of miR-371-3	Promotes neural differentiation		[47]
Downregulation of miR-132	Promotes the differentiation of tyrosine hydroxylase-positive neurons	Nurr1	[48]
miR-125	Promoted human ESC differentiation towards the neural lineage	Smad4	[49]
miR-302	Forms a regulation loop with *OCT4* during neural differentiation	Interacting with NR2F2	[50]
miR-138	Suppresses osteoblast differentiation of human MSCs	Targeting FAK and downstream signaling	[51]
miR-23b	Induces chondrogenic differentiation of hMSCs	By suppressing PKA signaling	[52]
miR-335-5p	Promotes the chondrogenic differentiation of mouse MSCs	Daam1 and ROCK1	[53]
miR-181	Promotes B-lymphocytes differentiation		[55]
miR-125a	Increases the number of HSCs	Bax1	[56]
miR-125b	Leads to a lethal myeloid leukemia in mice		[57]

miRNAs regulate the reprogramming, pluripotency, and differentiation of stem cells

## Additional files

Additional file 1: miRNAs regulate the process of induced reprogramming and direct reprogramming. The miR-290 family, miR-106b-25 cluster, miR-302 family, and miR-130/301/721 promote OSKM-induced reprogramming. However, the miR-30/let-7 family and miR-34a prevent this process. Moreover, the miR-302/367 cluster and miR-200c/302/369 cluster miRNAs could induce the reprogramming of somatic cells directly [26–34]. *OSKM* Oct4, Sox2, Klf4, c-myc; *OSK* Oct4, Sox2, Klf4; *MET* mesenchymal-to-epithlial transition; *iPSC* induced pluripotent stem cells. The *red arrows* indicate promotion, the *green suppression symbols* indicate inhibition. (PPTX 69 kb)

Additional file 2: The regulatory mechanisms of miRNAs in cardiovascular differentiation. miRNAs always target specific cardiovascular differentiation markers to modulate differentiation [37–43]. The *red arrows* indicate promotion, the *green suppression symbols* indicate inhibition. (PPTX 50 kb)

Additional file 3: miRNAs modulate neural differentiation signal pathways. Important regulatory mechanisms of miRNAs in neural differentiation [46–51]. *NSC* neural stem cell, *hESC* human embryonic stem cell, *hiPSC* human induced pluripotent stem cell. The *red arrows* indicate promotion, the *green suppression symbols* indicate inhibition. (PPTX 55 kb)

Additional file 4: miRNAs mediate osteogenic and chondrogenic differentiation. miRNAs mainly target the osteogenic and chondrogenic differentiation markers and signal pathways to regulate differentiation [52–54]. The *red arrows* indicate promotion, the *green suppression symbols* indicate inhibition. (PPTX 50 kb)

## Abbreviations

EC: Endothelial cell
ESC: Embryonic stem cell
ESCC: Embryonic stem cell-specific
FAK: Focal adhesion kinase
HSC: Hematopoietic stem cell
iPSC: Induced pluripotent stem cell
MET: Mesenchymal-to-epithlial transition
miRNA: microRNA
MSC: Mesenchymal stem cell
NSC: Neural stem cell
OSK: Oct4, Sox2, Klf4
OSKM: Oct4, Sox2, Klf4, c-Myc
PKA: Protein kinase A
pre-miRNA: Precursor miRNA
pri-miRNA: primary miRNA
SMC: Smooth muscle cell
UTR: Untranslated region.
