# Supplementary material for: microRNAs: important regulators of stem cells
Source: Stem Cell Res Ther. 2017 May 11;8:110. doi: 10.1186/s13287-017-0551-0 (PMC5426004; doi:10.1186/s13287-017-0551-0)
Supplement: Supplementary file 1 — miRNAs regulate the process of induced reprogramming and direct reprogramming. The miR-290 family, miR-106b-25 cluster, miR-302 family, and miR-130/301/721 promote OSKM-induced reprogramming. However, the miR-30/let-7 family and miR-34a prevent this process. Moreover, the miR-302/367 cluster and miR-200c/302/369 cluster miRNAs could induce the reprogramming of somatic cells directly [26–34]. OSKM Oct4, Sox2, Klf4, c-myc; OSK Oct4, Sox2, Klf4; MET mesenchymal-to-epithlial transition; iPSC induced pluripotent stem cells. The red arrows indicate promotion, the green suppression symbols indicate inhibition. (PPTX 69 kb) [file 13287_2017_551_MOESM1_ESM.pptx]

## Slide 1
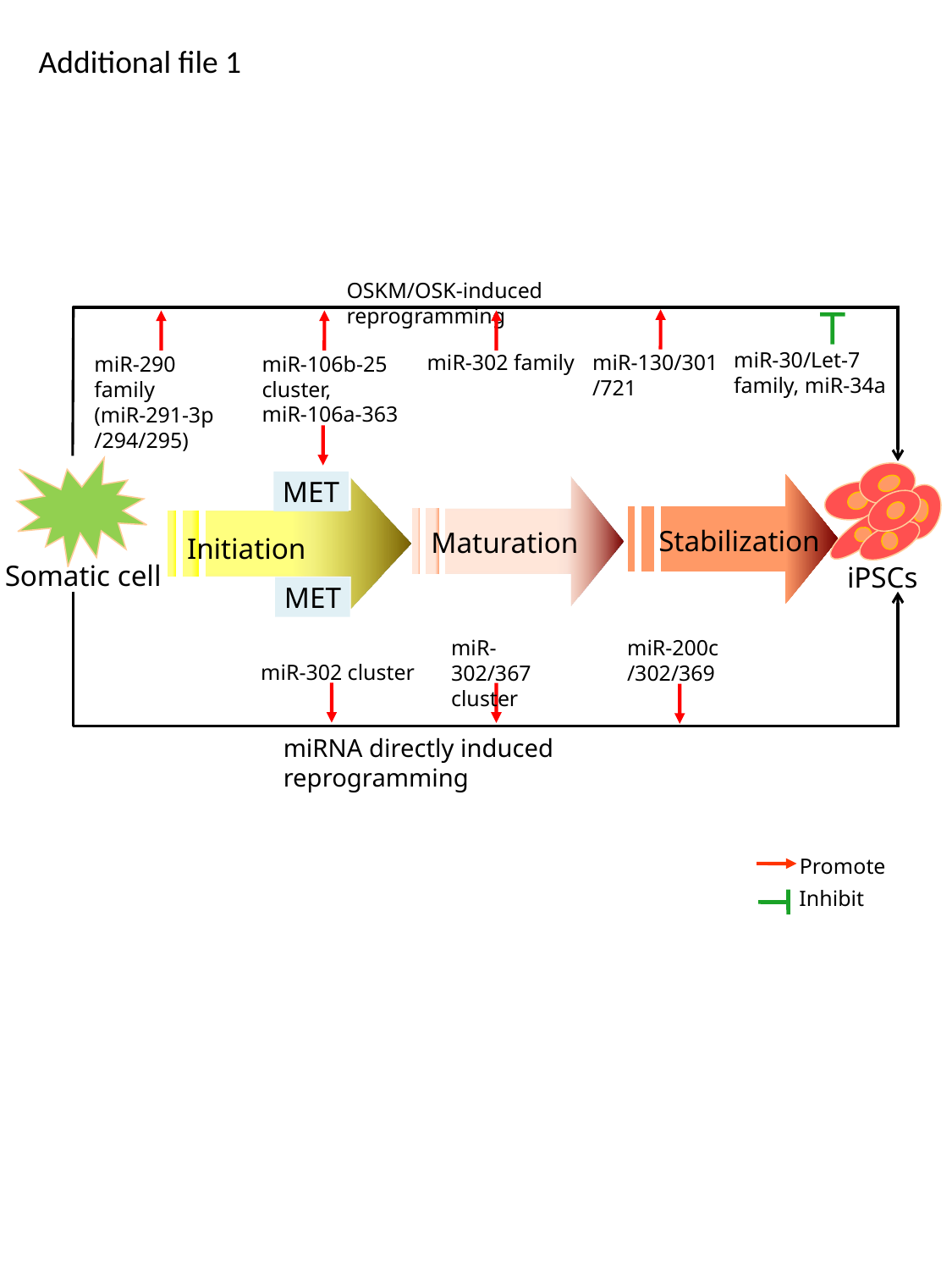

Additional file 1
OSKM/OSK-induced reprogramming
miR-30/Let-7
family, miR-34a
miR-130/301
/721
miR-302 family
miR-106b-25
cluster,
miR-106a-363
miR-290 family
(miR-291-3p
/294/295)
MET
Stabilization
Maturation
Initiation
Somatic cell
iPSCs
MET
miR-302/367
cluster
miR-200c
/302/369
miR-302 cluster
miRNA directly induced reprogramming
Promote
Inhibit
