# Supplementary material for: microRNAs: important regulators of stem cells
Source: Stem Cell Res Ther. 2017 May 11;8:110. doi: 10.1186/s13287-017-0551-0 (PMC5426004; doi:10.1186/s13287-017-0551-0)
Supplement: Supplementary file 2 — The regulatory mechanisms of miRNAs in cardiovascular differentiation. miRNAs always target specific cardiovascular differentiation markers to modulate differentiation [37–43]. The red arrows indicate promotion, the green suppression symbols indicate inhibition. (PPTX 50 kb) [file 13287_2017_551_MOESM2_ESM.pptx]

## Slide 1
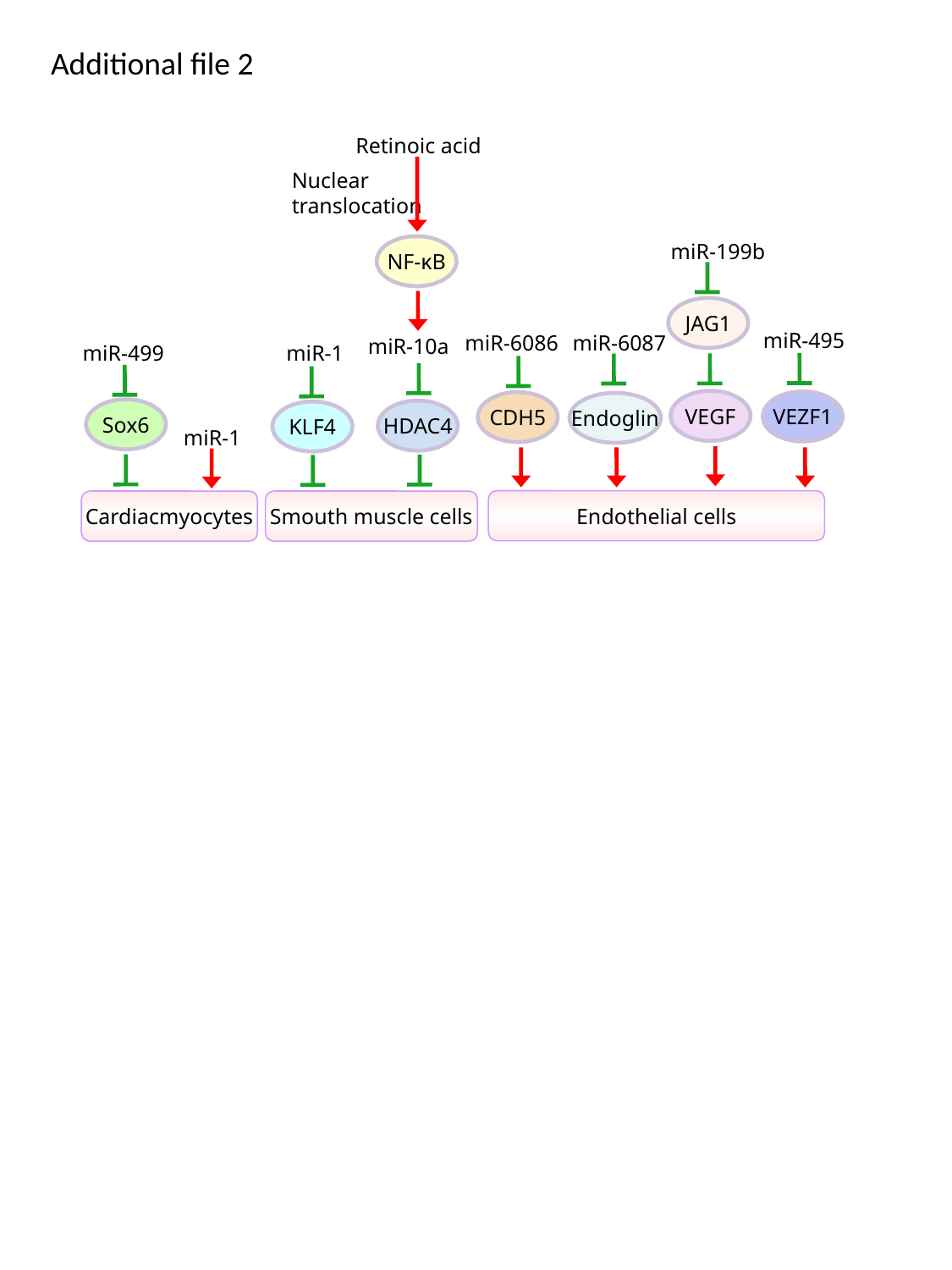

Additional file 2
Retinoic acid
Nuclear
translocation
miR-199b
NF-κB
JAG1
miR-495
miR-6086
miR-6087
miR-10a
miR-1
miR-499
VEGF
VEZF1
CDH5
Endoglin
Sox6
HDAC4
KLF4
miR-1
Endothelial cells
Cardiacmyocytes
Smouth muscle cells
