# Supplementary material for: microRNAs: important regulators of stem cells
Source: Stem Cell Res Ther. 2017 May 11;8:110. doi: 10.1186/s13287-017-0551-0 (PMC5426004; doi:10.1186/s13287-017-0551-0)
Supplement: Supplementary file 3 — miRNAs modulate neural differentiation signal pathways. Important regulatory mechanisms of miRNAs in neural differentiation [46–51]. NSC neural stem cell, hESC human embryonic stem cell, hiPSC human induced pluripotent stem cell. The red arrows indicate promotion, the green suppression symbols indicate inhibition. (PPTX 55 kb) [file 13287_2017_551_MOESM3_ESM.pptx]

## Slide 1
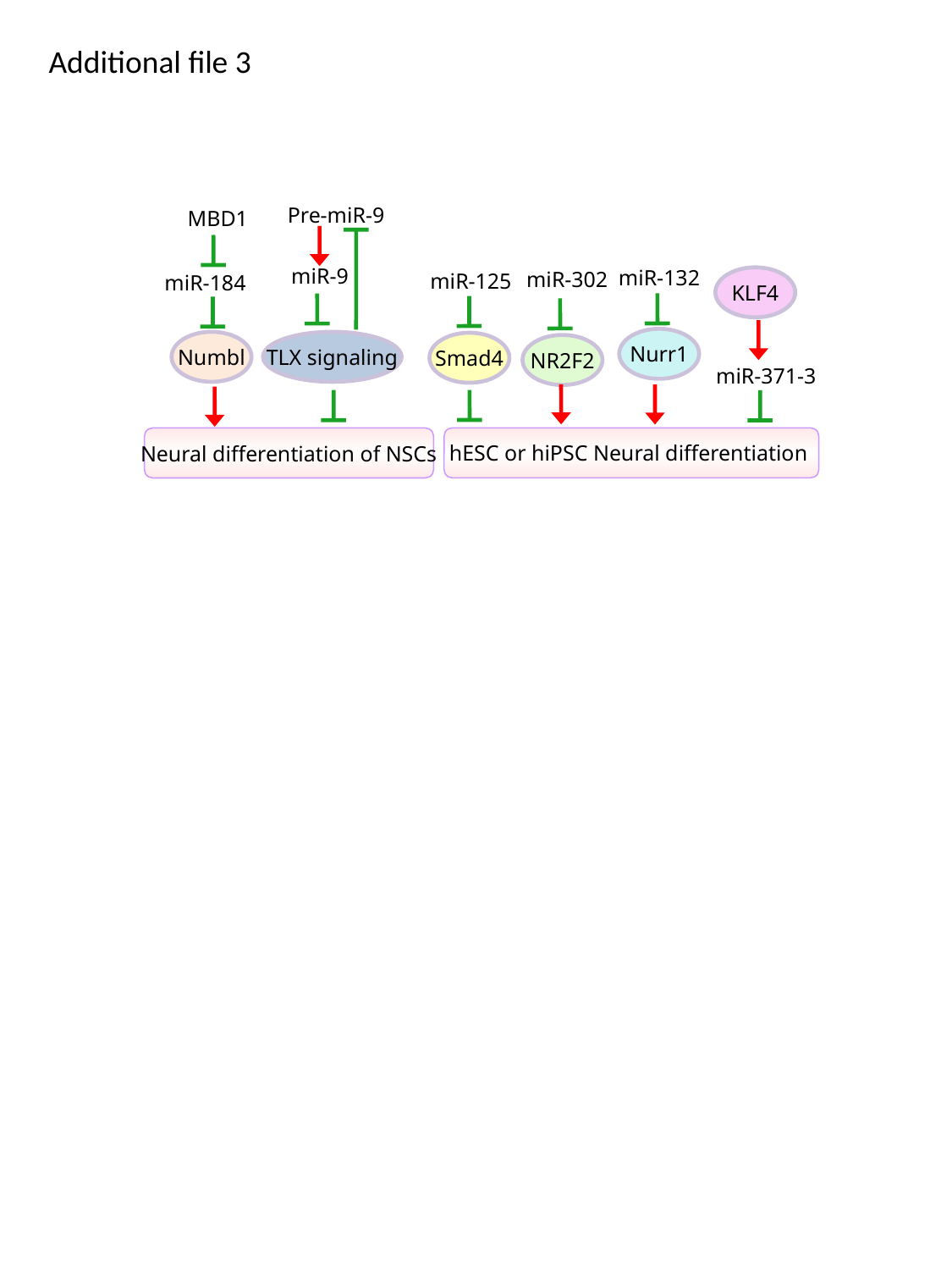

Additional file 3
Pre-miR-9
MBD1
miR-9
miR-132
miR-302
miR-125
miR-184
KLF4
Nurr1
TLX signaling
Numbl
Smad4
NR2F2
miR-371-3
hESC or hiPSC Neural differentiation
Neural differentiation of NSCs
