# Supplementary material for: microRNAs: important regulators of stem cells
Source: Stem Cell Res Ther. 2017 May 11;8:110. doi: 10.1186/s13287-017-0551-0 (PMC5426004; doi:10.1186/s13287-017-0551-0)
Supplement: Supplementary file 4 — miRNAs mediate osteogenic and chondrogenic differentiation. miRNAs mainly target the osteogenic and chondrogenic differentiation markers and signal pathways to regulate differentiation [52–54]. The red arrows indicate promotion, the green suppression symbols indicate inhibition. (PPTX 50 kb) [file 13287_2017_551_MOESM4_ESM.pptx]

## Slide 1
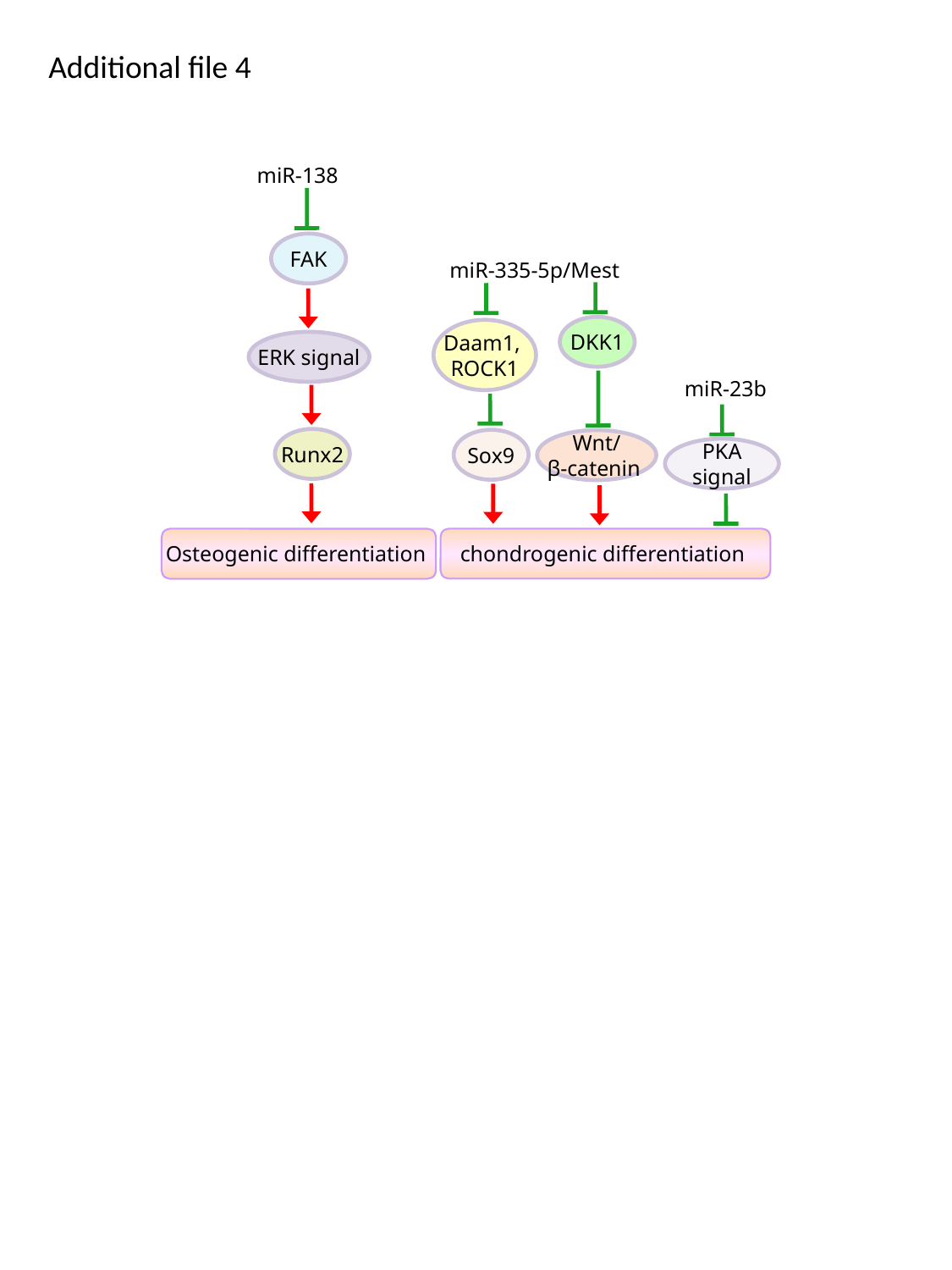

Additional file 4
miR-138
FAK
miR-335-5p/Mest
DKK1
Daam1,
ROCK1
ERK signal
miR-23b
Runx2
Sox9
Wnt/
β-catenin
PKA
signal
chondrogenic differentiation
Osteogenic differentiation
